# Mental health stigma at primary health care centres in Lebanon: qualitative study

**DOI:** 10.1186/s13033-022-00533-y

**Published:** 2022-05-07

**Authors:** Racha Abi Hana, Maguy Arnous, Eva Heim, Anaïs Aeschlimann, Mirja Koschorke, Randa S. Hamadeh, Graham Thornicroft, Brandon A. Kohrt, Marit Sijbrandij, Pim Cuijpers, Rabih El-Chammay

**Affiliations:** 1 Ministry of Public Health, National Mental Health Programme, Beirut, Lebanon; 2 Department of Clinical, Neuro- and Developmental Psychology, Vrije Universiteit, Amsterdam, The Netherlands; 3 Institute of Psychology, University of Lausanne, Lausanne, Switzerland; 4 Department of Psychology, University of Zurich, Zurich, Switzerland; 5 Centre for Global Mental Health and Centre for Implementation Science, Institute of Psychiatry, Psychology and Neuroscience, King’s College London, London, England; 6 Primary Healthcare Department at Ministry of Public Health, Beirut, Lebanon; 7 Global Health Team of Experts (GHTE), Beirut, Lebanon; 8 Department of Psychiatry and Behavioral Sciences, Division of Global Mental Health George Washington University, Washington, DC USA; 9 Department of Psychiatry, Saint Joseph University, Beirut, Lebanon

**Keywords:** Primary health care (PHC), Mental health Stigma, Qualitative research

## Abstract

**Background:**

Mental health-related stigma is a global public health concern and a major barrier to seeking care. In this study, we explored the role of stigma as a barrier to scaling up mental health services in primary health care (PHC) centres in Lebanon. We focused on the experiences of Healthcare Providers (HCPs) providing services to patients with mental health conditions (MHCs), the views of policy makers, and the perceptions of stigma or discrimination among individuals with MHCs. This study was conducted as part of INDIGO-PRIMARY, a larger multinational stigma reduction programme.

**Methods:**

Semi-structured qualitative interviews (n = 45) were carried out with policy makers (n = 3), PHC management (n = 4), PHC staff (n = 24), and service users (SUs) (n = 14) between August 2018 and September 2019. These interviews explored mental health knowledge, attitudes and behaviour of staff, challenges of providing treatment, and patient outcomes. All interviews were coded using NVivo and a thematic coding framework.

**Results:**

The results of this study are presented under three themes: (1) stigma at PHC level, (2) stigma outside PHC centres, and (3) structural stigma. SUs did not testify to discrimination from HCPs but did describe stigmatising behaviour from their families. Interestingly, at the PHC level, stigma reporting differed among staff according to a power gradient. Nurses and social workers did not explicitly report incidents of stigma but described patients with MHCs as uncooperative, underscoring their internalized negative views on mental health. General practitioners and directors were more outspoken than nurses regarding the challenges faced with mental health patients. Mental health professionals revealed that HCPs still hold implicitly negative views towards patients with MHCs however their attitude has improved recently. Our analysis highlights five layers of stigma affecting SUs.

**Conclusion:**

This qualitative study reveals that stigma was still a key concern that affects patients with MHC. SUs reported experiencing overt stigmatising behaviour in the community but less explicit discrimination in a PHC setting. Our findings emphasise the importance of (1) combatting structural stigma through legal reform, (2) addressing interpersonal stigma, (3) committing PHC management to deliver high quality mental health integrated services, and (4) reducing intrapersonal stigma by building public empathy.

## Background

Mental health-related stigma is a global public health concern. It is considered a major barrier to seeking care and ongoing treatment participation. Stigma may cause fear and reluctance to seek help amongst people with mental health conditions (MHCs) [1–3].

Stigma is an indication of shame that typically creates unfavourable attitudes towards the receiver, leading to derogatory discrimination when it is related to a person with a MHC [4].It is a heterogeneous notion including a spectrum of prejudicial perceptions, attitudes, and behaviours [5] related to a lack of knowledge that leads to ignorance and discrimination [4]. More specifically, stigma is divided into two categories of variants: “experiential variants”—including perceived, endorsed, anticipated, received, and enacted stigma—and “action-oriented variants”—including public, structural, provider-based and self-stigma [6] Stigma and discrimination connected to MHCs have been portrayed as having worse effects and causing more suffering than the mental health problems themselves [7]. Thus, addressing stigma has been highlighted as a significant objective of the World Health Organization (WHO) Action Plan 2013–2020 for mental health [8].

Within the service delivery context, stigma is understood to function at three interlinked levels: (1) structural stigma represented by policies and legislations, (2) interpersonal stigma constituted by issues related to knowledge, attitudes and behaviour of HCPs and one’s community, and (3) intrapersonal stigma connoted by negative beliefs about the self, including both self-stigma and internalized stigma [9]. Health professionals’ behaviour can affect self-stigma due to the effect of interpersonal interactions [9]; for example, experiences of self-stigma are amplified when health professionals describe MHCs with stigmatising or judgmental terms instead of actively attempting to understanding a person’s experience with their illness [10].

In general medical settings, research has shown that healthcare providers may express negative attitudes of fear, guilt and aggression towards patients with MHCs, which has a negative impact on patient care [11]. As such, stigma appears to be a barrier to receiving compassionate treatment, even when the patients’ primary reasons for admission are unrelated to pre-existing mental health conditions [11]. Some practitioners may hold stereotyped beliefs, so that they treat mental health patients negatively, and may label them and perceive them to be dangerous [12].

In low–and middle-income countries (LMICs), stigma and discrimination towards individuals with MHCs have led to a high prevalence of human rights violations (including basic cultural, civil, economic, political, and social rights) [13]. In Arab countries, stigma remains largely understudied [14] and continues to be a significant obstacle to adequate mental healthcare provision [15]. Another important problem affecting structural stigma, especially in LMICs, is the scarcity of resources in terms of funding, community resources and human resources.[16, 17]. Therefore, patients with MHCs may not receive appropriate or effective care for their mental or physical health due to poor staff training, inadequate supervision, and other structural factors [9].

Lebanon is a middle-income country with an overall treatment gap of more than ninety percent in mental health [18]. Lebanon is known for its political unrest. The health system was overstretched due to the increase of the population residing in Lebanon as a result of the Syrian crisis [19] Mental health resources in Lebanon are constrained despite the large need for mental health and psychosocial support services [20]. In 2014, the National Mental Health Programme (NMHP) was established with the aim of reforming the mental health framework and scaling up services. In reference to the first national strategy “Mental Health and Substance Use Prevention, Promotion, and Treatment Strategy for Lebanon 2015–2020”, stigma remains a main challenge in enhancing mental health service use [20]. Based on the findings precipitating the development of this national strategy, stigma is considered a cross-cutting encounter across all levels of care and is negatively impacting service development and delivery, as well as leading to discrimination [20]. The NMHP, in line with the national strategy, has been working with their partners to integrate mental health services into selected PHC centres by ensuring the availability of essential psychotropic medications and by providing mhGAP training and supervision for HCPs to be able to screen, assess, manage, or refer mental health cases when needed [20] However, this integration was subject to budget availability as well as to other structural challenges.

The aim of this study was to explore stigma associated with mental illness at primary health care (PHC) centres in Lebanon. It also aimed to inform a deeper understanding regarding the integration of mental health into PHC, which is a cornerstone of the mental health reform plan in Lebanon that started in 2015. This study thus intended to deepen our understanding of the experiences of PHC staff while providing services for patients with MHCs. Furthermore, the study aimed to understand the experiences of patients with MHCs when attending PHC centres, and whether they perceived any stigma or discrimination during their treatment. In addition, policy makers were included to provide context regarding the structure of Lebanon’s current healthcare system. This study was embedded within a larger programme, called INDIGO-PRIMARY, which seeks to develop an anti-stigma intervention resulting from cross-country findings that will support both staff and patients with MHCs in primary care [21].

This paper presents results from the situational analysis investigating current processes at PHC centres in Lebanon [22]. These data are significant for understanding what should be done to decrease stigma and, in turn, to improve mental health treatment and provide support for patients with MHC in PHC settings in Lebanon, where the number of Mental Health Professionals (MHPs) working in the public sector is relatively low.

## Methods

### Design

Methods comprised the analysis of qualitative data arising from interviews with policy makers, PHC management, PHC staff, service users (SUs) (n = 45). The ethics protocol was approved from Saint Joseph’s University Beirut (CEHDF 1193). This study was nested within a multinational study (INDIGO -PRIMARY) investigating mental health stigma in primary care in seven countries [22].

Qualitative interviews consisted of semi-structured interviews using five different topic guides developed through the INDIGO-PRIMARY steering group and tailored to the interviewee [22]. The topic guides explored: provider knowledge about MHCs; implicit and explicit attitudes and behaviour of staff towards SUs; burnout; provider clinical competence and quality of care; primary care staff training levels and training needs; challenges of providing treatment; patient outcomes including experiences of stigma and discrimination during treatment; and sociocultural factors that affect patient treatment (Table 1). The topic guides provided a list of topics, broad questions, and probes to be explored, whereby the phrasing of questions was locally adapted to Lebanon and accounted for cultural and contextual factors. All questionnaires were translated to Arabic; the terms were adjusted to remove any stigmatising expression using the NMHP glossary of Arabic mental health terminology. Non—stigmatising terminology was considered to reduce stigma and negative bias when talking about MHCs. Questionnaires were tested by NMHP staff internally and adapted accordingly.

**Table 1:** Areas included in the topic guides

Programme managers and policy makers • Health system structural and organisational factors
Lead primary care clinician or manager • Provision of mental health care at the PHC • Training and supervision for primary care providers (includes questions on PHC worker knowledge) • Potential barriers to optimal practice (includes questions on staff burnout, attitudes and clinical competence/quality of care) • Socio-cultural factors (includes questions on attitudes)
Primary care staff (clinical, administrative and support staff) in selected PHC centres • Description of the role and commonly reported mental health problems • Training and supervision for primary care providers (includes questions on PHC worker knowledge) • Potential barriers to optimal practice (includes questions on staff burnout, attitudes and clinical competence/quality of care) • Socio-cultural factors (includes questions on knowledge and attitudes)
Associated mental health professionals • Description of the role • Role in training and supervision of PHC staff and accepting referrals • Experiences of supporting primary care providers and challenges • Staff knowledge attitudes behaviour • Role in any anti-stigma training or anti-stigma efforts • Priority areas for interventions to address knowledge, attitudes and behaviours
Service users (SUs) • Description (age, socioeconomic, demographics) • Type of mental health problems, explanatory models, help-seeking and possible reasons for delays in helpseeking • Experiences with treatment • Experiences of stigma and discrimination • Resources and anti-stigma interventions

A member of the local research team explained the study to participants verbally and gave them an accompanying participant information sheet to read. All participants were asked to complete and sign an informed consent form at the beginning of the study except when the interview was conducted over the phone. The outreach process of participants was done through focal point staff of PHC centres, tasked with describing the study and obtaining the verbal consent of participants. The interviewer would reiterate the explanation of the study and the consent form to the participant when the phone call was scheduled. The interviewer made sure the participant had adequate time to ask clarifying questions before the interview. Phone interviews were mainly done for SUs who preferred so, and in these cases, consent was taken verbally.

The participant information sheet and consent form stated that participation in the study was voluntary, and participants could withdraw at any time, and explained the aims and nature of the study, as well as what was expected from participants, in lay terms.

### Participants

Selection of four PHC centres was done in collaboration with the PHC department at MoPH and the NMHP team. The PHC centres were selected based on the following criteria: (a) having staff trained on mhGAP, (b) having a high patient load to be able to interview an acceptable number of SUs, (c) availability of MHPs to be interviewed, and (d) location in Beirut and Mount Lebanon due to the convenience of these two urban areas. The NMHP team coordinated with the focal persons in each PHC centre (director of centres or management coordinators) who ensured the first contact with key informants and SUs, who were chosen according to availability and identification with one of our key stakeholder groups; no other specific sampling method was applied. In accordance with the cross-country INDIGO PRIMARY study, five categories of stakeholders were included in the sample.

The first category was Primary care providers (at least three participants per PHC centre), which included both clinical and administrative staff. In this category, two levels of providers and staff were interviewed. First-level providers were those who work within primary care centres and who had received general rather than specialist mental health training (i.e. mhGAP training). Cadres included were general practitioners, family doctors, nurses, and other general paraprofessionals. Second-level providers were the administrative and support staff working in the centres, who had direct or indirect contact with SUs.

The second category was SUs (at least three participants per PHC centre). Eligible participants were persons with a diagnosis of a MHC attending one of the participating primary care services and seeking care for themselves. SUs diagnosis was purposively not asked, so as to encourage frank disclosure by participants without fear that confidentiality might be breached. They had to be able to provide consent for taking part in the study and aged 18 years or older but of any gender and nationality. Participants whose current state of functioning inhibited their capacity to comprehend the study, provide consent and perform the research activities (as assessed by their primary care doctor or health worker), e.g. SUs in psychiatric emergencies, were excluded from participation.

The third category was primary care centre managers or lead primary care clinicians (at least one per PHC centre), i.e., the local lead for managing staff and services provided at the primary care facility.

The fourth category was affiliated MHPs (at least one per PHC centre): MHPs who collaborate with the primary care facility, e.g., psychiatrists or psychologists providing services at the PHC centre.

The fifth category was programme managers and policymakers (at least one): local or national representatives of health authorities, institutions involved in policymaking, or funding bodies relevant to primary mental healthcare.

### Data collection

Data were collected through qualitative interviews (n = 45), Programme Managers and Policy Makers (n = 3), SUs (n = 14), Nurses (n = 6), General Practitioners (GPs) (n = 5); Mental Health Professionals (n = 6), Frontline practitioners (n = 4), PHC Management (n = 4), and other staff (n = 3) (Table 2).

**Table 2:** Qualitative sample participant demographics

Stakeholder group	Number
***Primary care providers***	
Men	4
Women	13
Doctor	5
Nurse	6
Social worker	2
Receptionist/secretary/admin staff	4
Age 19–39	7
Age 40 or above	4
Age not known	6
No prior mental health training or experience	3
Any prior mental health training or experience	14
Not known	0
Total	17
***Lead primary care clinicians/managers***	
Men	2
Women	2
Age 19–39	0
Age 40 or above	4
Total	4
***Mental health professionals***	
Men	1
Women	6
Age 19–39	4
Age 40 or above	1
Age not known	2
Total	7
***Service users (SUs)***	
Men	2
Women	12
Common mental disorder	14
Severe mental disorder	0
Diagnosis not known	0
Age 19–39	7
Age 40 or above	7
Total	14
***Policy makers***	
Men	2
Women	1
Total	3
Grand total	45

The data collection phase was done in two stages. First, the NMHP team conducted 12 qualitative interviews with HCPs and SUs between August and December 2018 in two primary care centres. Then the team tried, unsuccessfully, to interview additional SUs from these two PHC centres. This was in fact a main challenge in the first round of interviews, where SUs either were not available since the MHP stopped working in one of the PHC centres or SUs refused to be interviewed or failed to show up to a scheduled meeting with the interviewer. Reasons for participants’ refusal were not disclosed to the research team by the PHC centre focal point staff.

The second stage expanded on the work done in phase one in order to gather more data from PHC centres through more interviews. Since the recruitment of SUs in the first round was challenging, we gave them the option to be interviewed over the phone to ensure greater privacy and convenience. These additional interviews were launched in September 2019 with new staff members in one of the previous PHC centres, as well as in two new PHC centres in Beirut and Mount Lebanon. In this phase, 31 individual interviews were conducted (for staff, SUs) in the PHC centres as well as three other interviews with policy makers and programme managers. All interviews were conducted in Arabic and were held for an average of approximately 30 min each. Findings for phase one of these interviews were included in the cross-country analysis [22], whereas this paper goes beyond this initial sample to cover the perspectives of a larger number of interviewees (n = 45).

### Data analysis

Interviews were recorded, transcribed, and translated to English by the NMHP team, then verified again in comparison to the recordings. All interviews were coded using NVivo and analysed using a thematic coding framework [23, 24]. The thematic coding framework was developed jointly by all researchers across all sites to frame multiple-researcher coding [22]. A code book had already been developed in Tunisia, which included the main topics in the interview guide and updates only to reflect minor differences in the Lebanon site. Consensus coding was used to reach inter-rater reliability: each coder independently coded each interview, then the two coders met to compare and agree on final codes. All codes were reviewed extensively by the project team to ensure accurate coding and removal of unnecessary dual coding.

## Results

The main results of this study are presented under three identified themes. The first theme is stigma at the PHC level and focuses on stigma as expressed by both SUs and by HCPs. The second theme is stigma outside PHC centres, as SUs experiences of stigma in the community, such as social exclusion on the basis of their condition, were more prominent and voiced openly by SUs. The third level is structural stigma at the system level.

### PHC level

#### Stigma as expressed by SUs

Across all PHC centres, SUs described positive experiences while accessing mental health services, including feeling accepted, respected, and well treated by the Healthcare Providers (HCPs). Experiences of stigma and discrimination within the selected PHC centres were never mentioned in 14 interviews with SUs from four PHC centres. The overall perception of staff behaviour from all PHC centres was positive in the way they treat and respond to patients with MHCs. Only one SU reported stigma in a PHC centre accessed prior to their current care; however, the SU did not want to discuss this issue further during their interview. Nevertheless, self-stigmatising behaviours and attitudes were voiced clearly in the aforementioned interviews. Self-stigma impacted SUs’ behaviour at the PHC centre; they described hiding their mental health conditions, their treatment with MHPs, or their dispositions from other HCPs and patients. According to one SU, self-stigma can also lead them to refrain from going to the PHC for treatment. One SU refused to acknowledge that he was receiving a mental health service as this might have suggested that he was insane. SUs belonging to vulnerable groups, such as members of the LGBTQI community and Syrian refugees, reported being respected and heard in the PHC centres with no incidents of discrimination mentioned. SUs made the point to contrast their positive experiences at the PHC centres with difficult and marked experiences of stigma and discrimination in Lebanon overall.

“Staff members at the centre are able to feel my pain. They understand my unhappiness.” (Service User1, PHC4, Female).

“I keep my condition private. I do not want anyone to know that I am seeing a psychiatrist.” (Service User5, PHC3, Female).

#### Stigma as expressed by healthcare providers

HCPs including nurses, receptionists and social workers did not explicitly report incidents of stigma or discrimination against patients with MHCs, but described the latter as violent, uncooperative or difficult, revealing internalized negative views on mental health and implicit stigma.

GPs and directors were more outspoken than nurses regarding the challenges faced when working with patients with MHCs. For them, they considered these patients to be a burden that PHC centres did not want to take on, continuously referencing accidents or crisis incidents caused by patients with MHCs to highlight their violent behaviour. In addition, prioritization of reaching target patient quotas seemed to be significant to PHC centre directors, so that doctors often overlooked mental health causes of patients’ symptoms in order to increase patient turnover. The general attitude expressed by doctors and managers towards patients with MHCs seemed to be overwhelmingly negative. One GP even reported that patients with MHCs need to be institutionalized, isolated from their community, and kept under the supervision of the NMHP or the Ministry of Public Health (MoPH), and not the PHC centre. A management coordinator reported that, in the past, security officers were often called in to manage and watch over patients with MHCs, but later stated that treatment of patients with MHCs has greatly improved. This improvement was largely associated with appointment of a mhGAP trained nurse as the focal person to communicate with patients with MHCs in order to increase their acceptance of treatment and support in their centre.

Although the lack of SU experiences of stigma and discrimination in the PHC centres was encouraging, interviews with MHPs underscored some negative attitudes by HCPs towards patients with MHCs. One MHP explained that although HCPs’ attitudes may not translate into actual behaviours towards patients with MHCs, their curiosity to learn more about their disorders, share personal identifiers and talk about experiences with patients broke patient confidentiality. However, MHPs mentioned that the attitude and behaviour of HCPs towards mental health SUs have improved in recent years, especially as PHC centres grew their involvement in advocacy campaigns conducted by the NMHP at the MoPH, as well as in other mental health projects. Therefore, full integration of mental health services at PHC centres will require a long-term roadmap.

“It is possible that a patient comes in and he is very irritated. He might shout at the staff and say obscene things, we immediately know that he is suffering from MH problems… Usually they are nervous, they might instigate a fight with anyone. You can’t say no to them.” (Data entry officer, PHC centre 1, Female).

“A patient once had an anger fit and started to break items at the centre. I lost two laptops along with their data; so I had to buy two new laptops. The patient did not wish to wait to see the doctor, so he broke the laptops. There was another incident, which involved another patient with mental disorders who came to collect insulin for his mother. The patient had requested more. This led to an argument. The pharmacist resigned as a result. The pharmacist had to request the support of the security officer for safety reasons. I lost the pharmacist and I lost two laptops.” (Director, PHC centre 4, Male).

### Stigma outside PHC centres

#### Stigma as expressed by SUs in the community

SUs were referred to MHPs by clinicians at the centres or decided to refer themselves. SUs reported they were hiding their mental health condition from their partners, children and families for fear of abandonment, stigmatising, or discontinuation of treatment. A brother of a female SU threw her antidepressants in the garbage when he learned that she was seeking help for her MHC. Another SU expressed that her husband was unaware of her MHC because she was afraid that he might leave her if he knew. It was obvious that the social context surrounding SUs was often unsupportive. SUs were either neglected or held responsible for their mental health problems.

In an attempt to underline the reasons behind the unsupportive familial and communal environment, MHPs reported that religion, or at least some aspects of religious practices and their intimate influence on people’s lives, often acted as a barrier for help-seeking or acceptance of diagnoses by the patients and their families. One nurse also reported that the surrounding environment of her PHC centre believed that lack of religious practice leads to MHCs. For these reasons, HCPs of PHC centres suggested that working with religious figures was a way to help bring the patient to the centre for treatment.

“My neighbours do not understand why my children are so loud and that my children suffer from mental health problems.” (Service User, PHC centre 4, Female).

“My husband tells me I am mentally ill and makes fun of me.” (Service User, PHC centre 4, Female).

“Some people assume that the patient is going through difficulties (or has depression) because the patient does not pray. Some religious people think that a person who has faith would not be affected by life’s trials and tribulations. They think that instead of seeing a psychiatrist the patient should pray”. (Nurse1, PHC centre 3, Female).

### Structural stigma

According to the interviews with policy makers, PHC directors and healthcare providers, the factors influencing stigma at a structural level were lack of mental health training, understaffing, lack of resources and lack of staff care that are mainly affected by funding. Policy makers find themselves facing two parallel challenges: a top—down approach, starting with advocating for and revising existing mental health laws and securing a budget for the NMHP, which is often overlooked in a country in economic and political turmoil with far higher presumed priorities. On the other hand, a bottom-up approach is hindered by the very limited resources provisioned to PHC centres, decreasing the impetus to add mental health services. Policy makers advocated for a system level approach, hinting that the entire care system needs to be reformed.

GPs and directors particularly focussed on structural barriers. They stated that the time invested in mental health patients, be it for the initial assessment and diagnosis or the frequently needed follow-ups, was time that could have been allocated to many other patients. HCPs need more time with mental health patients in order to effectively complete assessments and screening forms, and to identify their diagnosis, since they are not mental health specialists. In other terms, patient quotas per GP, and their association with financial support, deter them from taking on patients with MHCs, whose care is often more time-intensive. Many healthcare providers did not see their role extending to providing mental healthcare or engaging with beneficiaries with MHCs, clearly stating during interviews that working with mental health patients was not part of their job description. While nurses were ambitious and enthusiastic with regards to providing mental health services at the PHC centres as instructed, these services added to their existing tasks and increased their workload. Nevertheless, this was a managerial decision that could not be refused. Several nurses reported experiencing burnout from their work, exacerbated by a lack of support from their PHC centres. While some PHC centres agreed to grant one day off for staff care, other PHC centres were advocating for them to use their allocated days of leave if they needed to rest.

MHPs emphasized the need for further training on principles and guidelines when treating mental health patients. However, structural barriers go beyond the discontinuity of mental health trainings, which certainly affected the knowledge of healthcare providers and their skills to deal with patients with MHCs. These barriers also include the interrupted and insufficient supervision provided to HCPs by MHPs. One GP mentioned that with lack of supervision and no incentives for the additional tasks, doctors will not be motivated or confident in their abilities to provide mental healthcare. For instance, once one PHC centre lost their attending psychologist, who was also in charge of supervising all patients with MHCs, the unsupervised HCPs repeatedly failed to record any MHC symptoms in a very vulnerable population.

“In the past, supervisors from the MoPH used to offer service providers at PHC centres with a lot of support. Supervisors were providing essential support. We completed the trainings offered by the MoPH a long time ago. Service providers are applying what they know. No trainings are being given at PHC centres at the moment. Service providers might have forgotten the content of the trainings that they received. They have not received trainings in over two years. Supervision is very important”. (Program Coordinator, Male).

“When we asked PHC staff members to provide mental health services, most staff members objected because they did not consider offering mental health support to be part of their job description”. (Program Coordinator, Female).

“To be able to provide mental health at the level of primary care you need to have a system level approach, so this means that it goes way beyond just training and supervision”. (Policy Maker, Male)

**Fig. 1:**
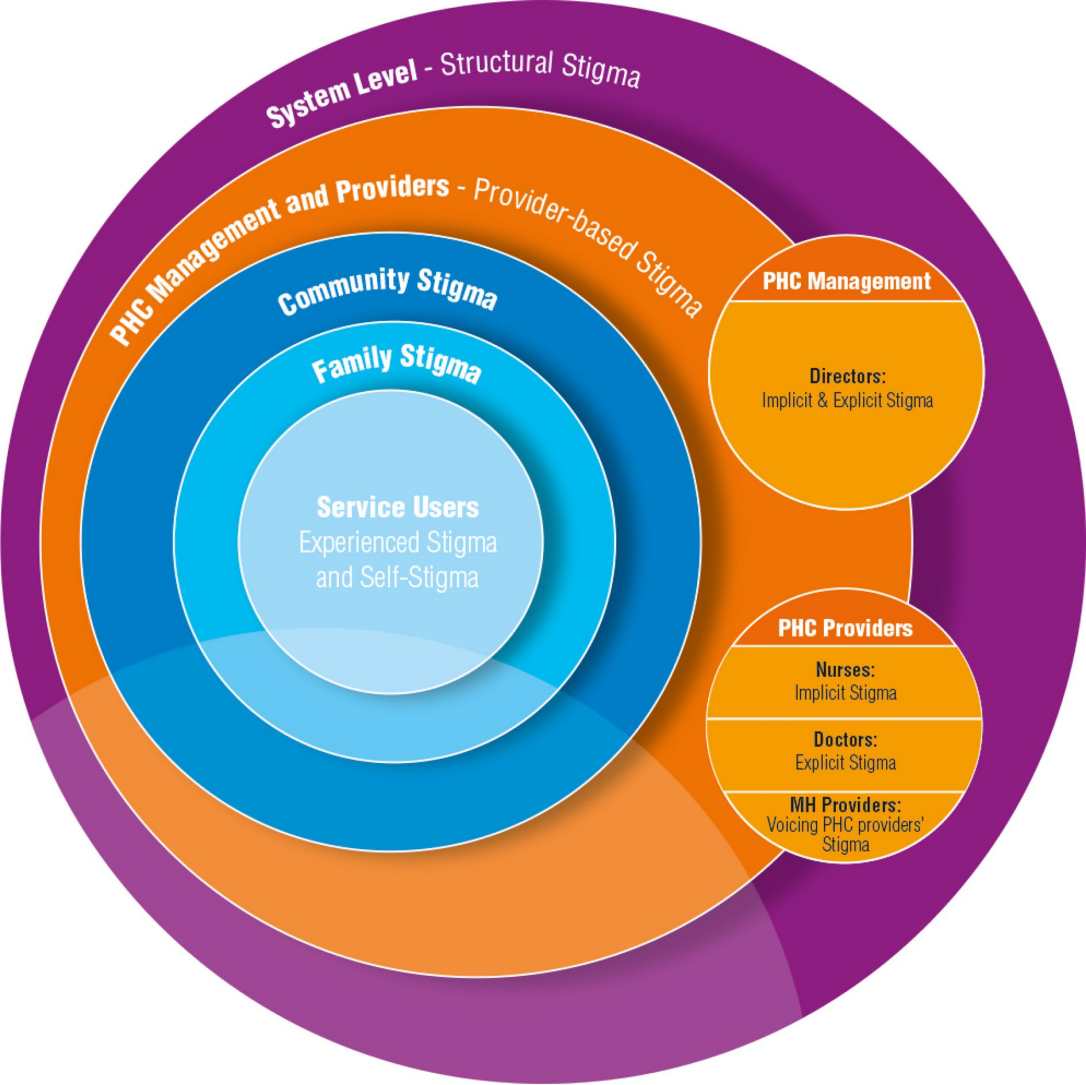
Results illustration divided into five layers of embedded stigma from the structural down to the self-level (Reference to the multilevel system layers of embedded stigma [6, 25])

Based on these findings, our main insights are interpreted in the following illustration (Fig. 1). Our analysis exposes five layers of stigma affecting people with MHCs: (1) structural, systemic stigma, (2) implicit and explicit provider-based stigma at the PHC level, (3) community stigma, (4) family stigma and (5) self-stigma. Interestingly, at the PHC level, our summary of findings and stigma reporting indicated differences in discussions based on a respondent’s place within the centre’s hierarchical structure, implying a possible link to inherent power differentials when disclosing such information.

## Discussion

### Community level

Although SUs did not report stigmatising attitudes at the PHC level, these findings should be subject to further investigation, because understanding the socioeconomic backgrounds and the dire needs of all SUs interviewed could explain the positive feedback we received from them. The fear of losing a free or semi-free support with all their medical related concerns could have influenced their answers, despite undergoing a consent process in which they were informed that care would not be affected by the responses they gave. In addition, another factor contributing to their exclusively positive descriptions of interactions with PHC staff could have been social desirability.

Koschorke and colleagues (2021) [22] provide recommendations to address these potential sources of biases when researching mental health stigma; they suggest not conducting qualitative interviews in clinical settings. Ideally, such interviews could be conducted in community settings, and persons with lived experience of MHCs could be trained to conduct the qualitative interviews. Another consideration is that PHC staff did not display discriminatory behaviour when the patients were present at the centre but waited the patients to leave and discussed internally their conditions and pointed on their behaviours.

The low level of stigma within healthcare centres, as reported by SUs, is also in line with a very recent study in Lebanon by Karam et al. [26] that investigated barriers to care through a sample of representative household adults: < 6% mentioned that stigma is a barrier to treatment. Rather, the greatest barrier to treatment is the low perceived need, as observed in 73.8% of the cases.

On the other hand, experiences of stigma in the community were prominent and voiced repeatedly by SUs. SUs felt unsupported by their community, families and partners as well, experiencing discrimination, hurtful labelling, and an internalization of negative beliefs about the self. These findings are in line with a recent study in Lebanon exploring cultural misconceptions about MHCs among Lebanese university students, [27] which found that 70% of the students believed that MHCs are taboo in Lebanese culture and should be hidden from family members. Several studies revealed widespread discrimination against patients with MHCs by family members, relatives, neighbours and the community throughout Arab countries, the MENA region, Asia and worldwide (e.g. [28–30]). Very few individuals in Arab countries are able to seek treatment from MHPs, and it may take years after onset before they seek care [31, 32]. In line with SUs’ reports and these aforementioned studies, it is clear that the discrimination from family members towards SUs would be an important topic for future research.

Other types of discriminatory behaviours linked to nationality, ethnicity, and sexual orientation are intimately linked to development of MHCs. In fact, since the start of the conflict in Syria, PHC centres in underdeveloped and marginalized areas became hubs of support for Syrian refugee families. With little and often ineffective work done on social cohesion between already underprivileged host-communities and the Syrian refugee communities [33, 34], the existing stigmatisation based on nationality, culture and traditions exacerbated the MHCs of these communities. Mental health problems were often reported to be directly linked to social determinants such as living conditions and poverty [35], as well as overt aggression from host communities (e.g., neighbours or employers) towards Syrian refugees and other ostracized communities (LGBTQI).

### PHC level

At the PHC level, many providers stated that recently stigma in society had diminished significantly due to increased knowledge and awareness around MHCs. This could be a result of increased activities on different fronts and from different MH actors to increase awareness and tackle stigma, including the work of the NMHP. However, it is important to continuously address further interpersonal stigma by increasing the level of awareness about mental health in the general public in Lebanon.

Contrasting reports from staff with those from MHPs indicated that stigma related to MHCs remains a barrier to care. A comparison between the interviews with nurses on the one hand and GPs on the other hand revealed that nurses did not openly describe events of discrimination against patients with MHCs, while doctors were more outspoken regarding this issue. They clearly expressed that patients with MHCs were a burden on the centre, and that their treatment should be handled by specialists or in institutionalised settings. The latter statement is in line with a study in Oman [36], where both medical students and the public preferred that psychiatric care facilities should be located away from the community.

These differences between nurses and GPs regarding their degree of open disclosure of stigma may be a by-product of their power and authority disparities, highlighting a pertinent power gradient in the medical field [37]. In most Arab countries, the literature shows that the reverence for physicians dominates over that for nurses, forming a weak image of nurses in Lebanon and the surrounding region [38]. Disclosing negative attitudes toward patients with MHCs may place nurses at greater risk for losing their jobs than physicians.

Primary care providers’ discussions of the challenges of treatment highlights implicit negative attitudes towards patients with MHCs. Some providers and managers shared such negative beliefs, such as believing that patients with MHCs are violent and thus deserving of blame for their conditions. According to the literature, this perspective has previously been seen to be a conceivable reaction to the misperception that MHCs are a personality weakness or that individuals are to be blamed for their disorder [39, 40]. Hence, this attitude may be considered an indication that service providers have misconceptions without voicing them explicitly, an assumption that should be further investigated. Consistent with findings by Ross and Goldner [11], the predominant attitude amongst nurses was that physical healthcare must be prioritized over mental healthcare, as it is seen as an additional burden to their job or was ‘not their job’. This attitude toward primary mental healthcare is likely aggravated by the lack of financial incentives.

In regard to interventions, awareness messages should be disseminated at the PHC level, particularly through evidence-based interventions. The existing evidence-base for high-income countries suggests that structured social contact between healthcare providers and persons with lived experience of MHCs has the potential to reduce stigma [41]. One strategy that has been piloted in LMICs is the Reducing Stigma among Healthcare Providers (RESHAPE) intervention in which people with lived experience are trained using Photo Voice to tell recovery stories. The visual recovery narratives presented by the persons with lived experience as well as other forms of structured social interaction of persons with lived experience are incorporated into mental health trainings for PHC staff. A pilot study showed that incorporating these social contact components into PHC trainings reduced stigma, increased MHC diagnostic accuracy of HCPs, and increased willingness to endorse and deliver psychological and psychosocial treatments (i.e., not solely rely upon medication) [42, 43]. The RESHAPE intervention is now being piloted in Tunisia, Ethiopia, India, and China. This work highlights that assessments of stigma should not be limited to only self-reported attitudes, but that behavioural assessments such as clinical role plays [44] and documentation of clinical services should also be evaluated during stigma interventions for HCPs.

### System level

As already mentioned, stigma can be implicitly found in nurses’ and physicians’ behaviour. However, this stigma can also be found explicitly within statewide policies [11]. A study by Reed and Fitzgerald [45] revealed that “mental healthcare may often be left till last, only carried out if there is still time, and only by those who feel able”. This also confirms that discriminatory policies and structural procedures prevent treatment seeking and funding for mental healthcare, therefore heavily influencing structural stigma [30, 39, 46, 47]. Similar findings regarding the overall importance of structural stigma in shaping other forms of stigma have been identified in other LMICs [48].

According to the interviews, training and supervision were highly emphasized as key to improving primary care providers’ knowledge on mental health and, in turn, to reducing stigma. This is consistent with the review conducted by van Boekel et al. [49], who highlighted that training, supervision and policies will improve structural factors and will have advantageous influences on the attitudes of HCPs. To intervene at a structural level, several factors need to be considered, including the revision of work policies, procedures, and job descriptions by PHC directors. Example initiatives for addressing structural factors include developing screening forms to identify mental health conditions and revising HCPs’ job descriptions to highlight their responsibilities in identifying and assessing mental health conditions. Other changes may include reforming PHC policies to include anti-stigma and confidentiality provisions as well as clinical and administrative mental health protocols. In addition, directors should also consider incentives for PHC staff. It is also important to ensure adequate infrastructure, such as the provision of secure storage of patient files for confidentiality and private rooms for consultation. Moreover, it is important to address the wellbeing of staff and provide self-care, since high stress levels can lead to burnout of HCPs [50]. Professional burnout has long been used as an explanation for stigmatisation in mental health care, and components of burnout, such as high emotional exhaustion and feelings of low personal accomplishment, have been shown to be significantly associated with negative attitudes toward mental health patients [9]. Ultimately, especially given the impact that COVID-19 had on mental health services, it is crucial to consider telemental health services as well as mobile-based intervention such as Step-by-Step, an e-mental health intervention by WHO and NMHP that was perceived to be relevant and acceptable [51]. In future research, it is important to evaluate whether newly implemented strategies, such as Step by Step, reduces stigma perceived by patients with MHC.

The establishment of the NMHP in 2014 and the launch of the first national strategy for mental health in 2015 [52] to reform the mental health system and scale up services have significantly expedited the provision of mental healthcare in PHCs. Although many changes have been enacted at the structural level of the health system in Lebanon [19], the total expenditure on mental health from the MoPH’s budget remains very minimal, and is mainly allocated for long-stay inpatient costs in mental hospitals [18, 53]. With the country facing economic collapse and political turmoil, funding challenges continue to inhibit the provision of adequate resources and training programmes, as well as interrupt the support and supervision provided by MHPs to HCPs.

### Strengths and limitations of the paper

This study included several trends within mental health that were previously understudied. To our knowledge, this is the first qualitative study in a PHC setting in Lebanon where SUs, along with a wide range of other stakeholders (n = 45), were interviewed about mental health-related stigma. Whereas in previous research the contribution of SUs had been ignored [4], in this study there was a relatively significant number of SUs (n = 14) which constituted around 30% of the total interviews. Furthermore, this study offered the ability to triangulate findings across a wide range of stakeholders and the capability to analyse results in a comprehensive approach. In addition, having variety and heterogeneity within the SUs sample enriched the study content and informed us of the diverse, heterogeneous perception of stigma and barriers related to the Lebanese context, since it included respondents who varied by gender, and nationality. Additionally, the present study also addressed structural stigma, which has not been studied in relation to stigma among primary care staff. An additional strength is that our qualitative approach allowed for an exploration of specific contexts and cultural indications of stigma which have not been studied previously. In reference to Evans-Lacko et al. [54], only 11% of previous studies on stigma selected a qualitative methodology, of which a majority lack a concise definition of stigma.

The study is not without its limitations. Firstly, it was conducted among four PHC centres in the Beirut and Mount Lebanon governorates only, for convenience of these areas, whereas other more underprivileged areas (such as North Lebanon, South Lebanon, Bekaa and rural areas) should be further explored in the future and may reflect other important outcomes. Another limitation is that SUs interview sample did not include people with severe mental illness, as in general, PHC centres are expected to treat people with less severe illness, nevertheless it is the case that occasionally PHC staff are asked to see people with severe mental illness due to their emergent nature. Furthermore, most of our findings rely on self-reports from PHC staff. Contact with most staff members (front liners, social workers, nurses & GPs) were exclusively established through their supervisors. SUs were also reached by contacting PHC centres focal points, who then referred SUs to the research team for interviews. This outreaching technique might have impacted the results due to responder bias. Although every effort was made to ensure confidentiality and assure SUs that their statements would not affect their treatment, the fear of losing services might still have affected some participants’ responses. A more direct sampling technique without going through the PHC centres may have reduced the risk of this bias occurring. Another aspect that would impact results is the possibility of social desirability bias and the attempt of interviewees to avoid painting themselves in an unfavourable light. Furthermore, previous collaborations and relations between the NMHP and PHC service providers may have reinforced the effect of social desirability. Finally, this study portrayed a period before Lebanon's intersecting crises, including its economic crisis, COVID-19 epidemic, political unrest, and the Beirut port blast, which had a significant impact on many sectors, including healthcare and mental health services in the country. As a result, the situation presently may now differ from that which existed prior to 2019.

### Implications of the findings

This study may inform future interventions at the primary care level and will inform mental health training programmes. It also provides qualitative findings that support the framework for bridging the mental health treatment gap at PHC centres, and in turn improving the integration of mental health services into primary care. Future activities and further analysis of the interviews can also be used to explore facilitators and barriers to integrating mental health into primary care and suggest interventions that can support this integration. In future research, ethnographic and direct observational data within PHC settings can complement interview findings. Furthermore, combining qualitative with quantitative methods may be useful for better understanding stigma and reaching a larger sample, which could be compared to studies within other settings in the MENA region and worldwide. In addition, more detailed information about the interaction between different types of stigma within various levels in a health system may shed light on the root causes of our findings. Future studies should examine the cultural and contextual factors informing stigma in primary care with larger study samples and in different areas to develop specific guidelines for cultural adaptations [55]. These findings can be used to inform adaptations of strategies to reduce MHC stigma among HCPs [5].

## Conclusion

Our qualitative study shows that stigma is a key concern affecting patients with MHC. SUs reported experiencing overt stigmatising behaviour in the community, but less explicit discrimination in a PHC setting. Interviews with MHPs, however, revealed that negative attitudes towards patients with MHC still exist implicitly within HCPs. Therefore, in order to decrease stigma and improve the quality of mental health treatment in these settings, our findings suggest new recommendations to tackle all layers of embedded stigma. First, structural stigma should be addressed by revising mental health laws, ensuring proper funding, increasing human resources, and changing policies to integrate MH at primary care settings. Second, interpersonal stigma should be tackled by providing continual support and supervision as well as regularly building the capacity of healthcare staff. Third, management officials at PHC centres are invited to commit to delivering high-quality integrated mental health services, and to give greater emphasis to staff care and performance-based incentives. Fourth, we propose development of initiatives to address intrapersonal stigma by building public empathy and enhancing capacity at both the individual and community level, with specific emphasis on PHC beneficiaries. Finally, and building on the findings of this study, we urge implementation of new interventions to reduce stigma at each discussed level [7, 45, 56, 57], in an effort to holistically bridge the mental health treatment gap.

## Data Availability

All data generated or analysed during this study are included in this published article and its supplementary information files. Mirja Koschorke, Nathalie Oexle, Uta Ouali, Anish V Cherian, Vayankarappadam Deepika, Gurucharan Bhaskar Mendon, Dristy Gurung, Lucie Kondratova, Matyas Muller, Mariangela Lanfredi, Antonio Lasalvia, Andrea Bodrogi Anna Nyulászi, Mario Tomasini, Rabih El Chammay, Racha Abi Hana, Yosra Zgueb, Fethi Nacef, Eva Heim, Anaïs Aeschlimann, Sally Souraya, Maria Milenova, Nadja van Ginneken, Graham Thornicroft, Brandon A. Kohrt, Perspectives of healthcare providers, SUs, and their family members about mental illness stigma in primary care settings: A multi—site qualitative study of seven countries in Africa, Asia, and Europe, PLOS One, 2021 [22].

## Abbreviations

MoPH: Ministry of Public Health
NMHP: National Mental Health Programme
PHC: Primary health care
MHC: Mental health condition
mhGAP: Mental health gap action programme
LMIC: Low and middle income countries
SUs: Service users
GPs: General practitioner
HCPs: Healthcare providers
MHPs: Mental health professionals
RESHAPE: Reducing Stigma among Healthcare Providers

## References

[1] Schomerus G, Angermeyer MC (2008). Stigma and its impact on help—seeking for mental disorders: what do we know?. Epidemiol Psichiatr Soc.

[2] Corrigan P (2004). How stigma interferes with mental health care. Am Psychol.

[3] Thornicroft G (2008). Stigma and discrimination limit access to mental health care. Epidemiol Psichiatr Soc.

[4] Thornicroft G, Rose D, Kassam A, Sartorius N (2007). Stigma: ignorance, prejudice or discrimination?. Br J Psychiatry.

[5] Kohrt BA, Turner EL, Jordans MJ (2020). Reducing mental illness stigma in healthcare settings: proof of concept for a social contact intervention to address what matters most for primary care providers. Soc Sci Med.

[6] Pescosolido BA, Martin JK (2015). The stigma complex. Ann Rev Sociol.

[7] Thornicroft G, Mehta N, Clement S, Evans-Lacko S, Doherty M, Rose D, Henderson C (2016). Evidence for effective interventions to reduce mental-health-related stigma and discrimination. Lancet.

[8] World Health Organization (2013). Mental health action plan 2013–2020. Issue Ment Health Nurs.

[9] Henderson C, Noblett J, Parke H, Clement S, Caffrey A, Gale-Grant O, Thornicroft G (2014). Mental health-related stigma in health care and mental health-care settings. Lancet Psychiatry.

[10] Sartorius N (2007). Stigma and mental health. Lancet.

[11] Ross CA, Goldner EM (2009). Stigma, negative attitudes and discrimination towards mental illness within the nursing profession: a review of the literature. J Psychiatr Ment Health Nurs.

[12] Link BG, Phelan JC (2001). Conceptualizing stigma. Ann Rev Sociol.

[13] Drew N, Funk M, Tang S, Lamichhane J, Chávez E, Katontoka S, Saraceno B (2011). Human rights violations of people with mental and psychosocial disabilities: an unresolved global crisis. Lancet.

[14] Ouali U, Jomli R, Nefzi R, Ouertani H, Nacef F (2017). Social stigma in severe mental illness in tunisia: clinical and socio—demographic correlates. Eur Psychiatry..

[15] Gearing RE, Al-Krenawi A (2012). Adaptation and translation of mental health interventions in Middle Eastern ARAB countries: a systematic review of barriers. Int J Soc Psychiatry.

[16] Thornicroft G, Chatterji S, Evans-Lacko S, Gruber M, Sampson N, Aguilar-Gaxiola S, Kessler RC (2017). Undertreatment of people with major depressive disorder in 21 countries. Br J Psychiatry.

[17] Saxena S, Thornicroft G, Knapp M, Whiteford H (2007). Resources for mental health: scarcity, inequity, and inefficiency. Lancet.

[18] World Health Organization (2015). WHO-AIMS report on mental health system in lebanon.

[19] Chammay R, Karam E, Ammar W (2016). Mental health reform in Lebanon and the Syrian crisis. Lancet Psychiatry.

[20] Ministry of Public Health (2015). Mental health and substance use- prevention, promotion, and treatment—situation analysis and strategy for Lebanon 2015–2020 Version 1.1.

[21] Thornicroft G, Bakolis I, Evans-Lacko S, Gronholm P, Henderson C, Kohrt BA, Sartorius N (2019). Key lessons learned from the indigo global network on mental health related stigma and discrimination. World Psychiatry.

[22] Koschorke Mirja, Oexle Nathalie, Ouali Uta, Cherian Anish V, Thornicroft Graham, Kohrt Brandon A (2021). Perspectives of healthcare providers, service users, and their family members about mental illness stigma in primary care settings: a multi—site qualitative study of seven countries in Africa, Asia, and Europe. PLOS ONE.

[23] Green J (2004). Book review: qualitative methods and health policy research. Sociol Res Online.

[24] Srivastava P, Hopwood N (2009). A practical iterative framework for qualitative data analysis. Int J Qual Methods.

[25] Cook JE, Purdie-Vaughns V, Meyer IH, Busch JT (2014). Intervening within and across levels: a multilevel approach to stigma and public health. Soc Sci Med.

[26] Karam EG, Karam GE, Farhat C, Thornicroft G (2018). Determinants of treatment of mental disorders in Lebanon: barriers to treatment and changing patterns of service use. Epidemiol Psychiatric Sci.

[27] Rayan A, Fawaz M (2017). Cultural misconceptions and public stigma against mental illness among lebanese university students. Perspect Psychiatr Care.

[28] Lauber C, Rössler W (2007). Stigma towards people with mental illness in developing countries in Asia. Int Rev Psychiatry.

[29] Sewilam AM, Watson AM, Kassem AM, Clifton S, McDonald MC, Lipski R, Nimgaonkar VL (2014). Suggested avenues to reduce the stigma of mental illness in the Middle East. Int J Soc Psychiatry.

[30] Stier A, Hinshaw SP (2007). Explicit and implicit stigma against individuals with mental illness. Aust Psychol.

[31] Eapen V, Ghubash R (2004). Help–seeking for mental health problems of children: preferences and attitudes in the United Arab Emirates. Psychol Rep.

[32] Dardas LA, Simmons LA (2015). The stigma of mental illness in Arab Families: a concept analysis. J Psychiatr Ment Health Nurs.

[33] Mourad, L., & Piron, L. (2016). Municipal service delivery, stability, social cohesion and legitimacy in Lebanon. An Analytical Literature Review. http://www.activearabvoices.org/uploads/8/0/8/4/80849840/municipal_service_delivery_stability_soc.pdf. Accessed 13 Oct 2021.

[34] World Vision International. (2015). Social cohesion between Syrian refugees and Urban host communities in Lebanon and Jordan. https://www.wvi.org/sites/default/files/World%20Vision%20International%20DM2020%20Social%20Cohesion%20Report.pdf. Accessed 13 Oct 2021.

[35] World Health Organization and Calouste Gulbenkian Foundation (2014). 2014.

[36] Al-Adawi S, Dorvlo AS, Al-Ismaily SS, Al-Ghafry DA, Al-Noobi BZ, Al-Salmi A, Chand SP (2002). Perception of and attitude towards mental illness in Oman. Int J Soc Psychiatry.

[37] El-Jardali F, Longuenesse E, Jamal D, Jabbour S, Giacaman R, Khawaja M (2012). Kronfol NM (2014): The public health workforce and human distributions for health. Public health in the Arab World.

[38] El-Jardali (2012). The public health workforce and human distributions for health.

[39] Corrigan P (2005). On the stigma of mental illness: practical strategies for research and social change. Am Psychol.

[40] Rüsch N, Todd AR, Bodenhausen GV, Corrigan PW (2010). Biogenetic models of psychopathology, implicit guilt, and mental illness stigma. Psychiatry Res.

[41] Knaak S, Modgill G, Patten SB (2014). Key ingredients of anti-stigma programs for health care providers: a data synthesis of evaluative studies. Can J Psychiatry.

[42] Kohrt BA, Jordans MJD, Turner EL (2021). Collaboration with people with lived experience of mental illness to reduce stigma and improve primary care services: a pilot cluster randomized clinical trial. JAMA Netw Open..

[43] Bhardwaj A, Gurung D, Rai S, Kaiser BN, Cafaro CL, Sikkema KJ, Lund C, Luitel NP, Kohrt BA (2022). Treatment preferences for pharmacological versus psychological interventions among primary care providers in nepal: mixed methods analysis of a pilot cluster rando. Int J Environ Res Public Health.

[44] Kohrt BA, Mutamba BB, Luitel NP, Gwaikolo W, Onyango Mangen P, Nakku J, Rose K, Cooper J, Jordans MJD, Baingana F (2018). How competent are non-specialists trained to integrate mental health services in primary care? Global health perspectives from Uganda, Liber. Int Rev Psychiatry.

[45] Reed F, Fitzgerald L (2005). The mixed attitudes of nurse's to caring for people with mental illness in a rural general hospital. Int J Ment Health Nurs.

[46] Hinshaw S (2006). The mark of shame: stigma of mental illness and an agenda for change.

[47] Holmes EP, Corrigan PW, Williams P, Canar J, Kubiak MA (1999). Changing attitudes about schizophrenia. Schizophr Bull.

[48] Gurung D, Poudyal A, Wang YL, Neupane M, Bhattarai K, Wahid SS, Aryal S, Heim E, Gronholm P, Thornicroft G, Kohrt B (2022). Stigma against mental health disorders in Nepal conceptualised with a 'what matters most' framework: a. Epidemiol Psychiatr Sci.

[49] Van Boekel L, C.Garretsen, H. F.  (2013). Stigma among health professionals towards patients with substance use disorders and its consequences for healthcare delivery: systematic review. Drug Alcohol Depend.

[50] Selamu M, Thornicroft G, Fekadu A, Hanlon C (2017). Conceptualisation of Job–related wellbeing, stress and burnout among healthcare workers in rural ethiopia: a qualitative study. BMC Health Serv Res.

[51] Heim E, Ramia JA, Hana RA, Van't Hof E (2021). Step-by-step: feasibility randomised controlled trial of a mobile—based intervention for depression among populations affected by adversity in Lebanon. Internet interv.

[52] Chammay R (2016). Reforming mental health in Lebanon amid refugee crises. Bull World Health Organ.

[53] Hijazi Z, Weissbecker I, Chammay R (2011). The integration of mental health into primary health care in Lebanon. Intervention..

[54] Evans-Lacko S, Courtin E, Fiorillo A, Thornicroft G (2014). The state of the art in European research on reducing social exclusion and stigma related to mental health: a systematic mapping of the literature. Eur Psychiatry..

[55] Heim E, Kohrt BA, Koschorke M, Milenova M, Thornicroft G (2018). Reducing mental health–related stigma in primary health care settings in low–and middle-income countries: a systematic review. Epidemiol Psychiatr Sci.

[56] Gronholm PC, Henderson C, Deb T, Thornicroft G (2017). Interventions to reduce discrimination and stigma: the state of the art. Soc Psychiatry Psychiatr Epidemiol.

[57] Rao D, Elshafei A, Nguyen M, Hatzenbuehler ML, Frey S, Go VF (2019). A systematic review of multi–level stigma interventions: state of the science and future directions. BMC Med.

